# Is Accessing Dental Care Becoming More Difficult? Evidence from Canada's Middle-Income Population

**DOI:** 10.1371/journal.pone.0057377

**Published:** 2013-02-20

**Authors:** Chantel Ramraj, Laleh Sadeghi, Herenia P. Lawrence, Laura Dempster, Carlos Quiñonez

**Affiliations:** Discipline of Dental Public Health, Faculty of Dentistry, University of Toronto, Toronto, Ontario, Canada; University of Florida, United States of America

## Abstract

**Objective:**

To explore trends in access to dental care among middle-income Canadians.

**Methods:**

A secondary data analysis of six Canadian surveys that collected information on dental insurance coverage, cost-barriers to dental care, and out-of-pocket expenditures for dental care was conducted for select years from 1978 to 2009. Descriptive analyses were used to outline and compare trends among middle-income Canadians with other levels of income as well as national averages.

**Results:**

By 2009, middle-income Canadians had the lowest levels of dental insurance coverage (48.7%) compared to all other income groups. They reported the greatest increase in cost-barriers to dental care, from 12.6% in 1996 to 34.1% by 2009. Middle-income Canadians had the largest rise in out-of-pocket expenditures for dental care since 1978.

**Conclusions:**

This study suggests that affordability issues in accessing dental care are no longer just a problem for the lowest income groups in Canada, but are now impacting middle-income earners as a consequence of their lack of, or decreased access to, comprehensive dental insurance.

## Introduction

In Canada, recent data have shown that challenges in accessing dental care no longer just affect those with no or low incomes, but also those above them on the economic ladder [1]–[5]. In this regard, specific attention has been placed on working poor Canadians, namely those that do not qualify for public dental care programs, yet generally do not have jobs that offer employment-based dental insurance, which is the country's dominant form of financing dental care for individuals and families [1]–[5]. It has also been anecdotally reported that in light of the recent global economic downturn, middle-income families are now contacting local public health agencies in an effort to access public dental care programs [1].

These challenges in accessing dental care are fundamentally linked to issues of affordability. In Canada, income and insurance are known to be the dominant predictors of the utilization of and access to dental care, specifically because of their ability to mitigate the costs of care [2], [6], [7], [8]. Yet evidence has shown that dental insurance plans have changed dramatically over the last 20 years and have been diminished in terms of both their quality and availability [1]. For example, the quality of dental insurance plans decreased following a recession in the 1990s, as Canadian firms found ways to cost-contain in part by altering benefit plans through the limiting of annual maximums and/or services, and/or through the introduction or expansion of deductibles, co-insurance or co-payments [1], [9], [10]. At the same time, the availability of employment-based dental insurance decreased as Canadian firms began to change the nature of employment by increasing part-time and temporary positions, thus limiting the growth of unionization [1]. Importantly, it is full-time, permanent, unionized jobs in large firms that have benefitted the most from the presence of dental insurance, and it is low- and middle-income employees that have observed the greatest impacts to their workplace structures in this way [1], [11], [12], [13]. Even in the United States, employment-based dental insurance among lower-middle income Americans declined from 80.2% in 1999 to 77.4% by 2002 [14].

Additionally, the costs of and demand for dental care have increased sharply in Canada [15], while incomes have remained nearly stagnant over the past 25 years [16]. When adjusting for inflation, total per capita dental care expenditures rose from approximately $6 in 1960 to close to $50 by 2008 ($360 in current dollars), an increase of approximately 730% [15]. As a measure of demand, the utilization of dental care rose from 49.5%, as reported in the Nutrition Canada National Survey (1970–1972), to 74.5%, as reported in the recent Canadian Health Measures Survey (2007–2009) [15]. At the same time, family earnings stagnated or declined for those at the bottom of the income distribution, while rising substantially at the top [16].

Overall then, with changes to insurance, incomes, the costs of dental care, and a greater demand for it, there is a reasonable case for the anecdotal reports of middle-income Canadians facing greater challenges in accessing dental care. As a result, this study explored the issue of access to dental care among middle-income Canadians, by outlining trends in: self-reported dental insurance coverage; self-reported cost-barriers to dental care; and out-of-pocket expenditures for dental care.

## Methods

### Study design and samples

This was a secondary data analysis of a series of surveys provided by Statistics Canada: the Canadian Health Measures Survey (CHMS), Canadian Community Health Surveys (CCHS), National Population Health Surveys (NPHS), General Social Surveys (GSS), Surveys of Family Expenditures/Health Expenditures (FAMEX/SHS), and Workplace and Employee Surveys (WES). These surveys were accessed online through the University of Toronto's Data Library, or through Statistics Canada's Research Data Centre (RDC) at the University of Toronto. Due to the nature of each survey's sample population, the current study was limited to Canadians aged 12 years of age and older living in private households across the country. The overall period of observation extended from 1978 to 2009.

- The Canadian Health Measure Survey (CHMS) was conducted from March 2007 to February 2009 and collected health measures, including oral health measures, from approximately 5,600 people, representing 97% of the Canadian population between 6 and 79 years of age [15].
- The Canadian Community Health Surveys (CCHS) are a series of cross-sectional surveys collecting information on the health status, health care utilization and health determinants of Canadians. Data are collected from Canadians aged 12 years of age and older; a sample of 65,000 respondents is required on an annual basis representing 98% of the Canadian population [17]. Data for the years 2001, 2003, 2005 and 2007 were used.
- The National Population Health Surveys (NPHS) collect information on the health status and health care utilization of the Canadian population and includes persons from all ages. These studies used files of the cross-sectional components in 1996/1997 (N = 73,402) and 1998/1999 (N = 15,249).
- The General Social Surveys (GSS) collect information on social support and living conditions over time. Data on dental care were collected in 1985 (N = 11,200) and 1991 (N = 11,924).
- The Surveys of Family Expenditures/Health Expenditures (FAMEX/SHS) record detailed annual spending patterns for a nationally and regionally representative sample of private households in Canada. Data from the FAMEX/SHS from 1978 (N = 9,356 households) to 2009 (N = 16,758 households) were used.
- The Workplace and Employee Surveys (WES) have collected data annually from 1999, encompassing both longitudinal and cross-sectional components. Employer side data of the cross-sectional components from 1999 (N = 6,322) to 2006 (N = 6,312) were used.

### Data variables and analysis

Self-reported dental insurance coverage, cost-barriers to dental care and out-of-pocket expenditures were used as proxies for access to care. All were checked for consistency within and between each survey. Wherever possible, inconsistencies in categorization were minimized by recoding the original variable into a reference set of variables adopted from the CHMS. The middle-income group was defined by using ‘income adequacy,’ which takes into account the total household income of a family and the number of people living in that household [15]. This study used Statistics Canada's definition of middle-income for the years 1994-2009, which includes households with: one or two people earning $15,000–$29,999, three or four people earning $20,000–39,999 and 5 or more people earning $30,000–$59,999. Since this information was not available prior to 1994, middle-income adequacy was calculated by converting the original income brackets to current dollars for each year prior to 1994 using the Consumer Price Index (CPI) of Canada. The CPI is the most widely used index for household or family incomes, reflecting average spending patterns by consumers in Canada. The annual CPI from 1971 to 2010 is reported by Statistics Canada. The year 2002 was chosen as the base year as it was the median year in the observation period. To convert current dollars of any year to constant dollars, the amount was multiplied by the index of the base year divided by the index of the chosen year [18]. For example, $10,000 in 1997 (CPI of 90.4) would be $11,602 in 2002 dollars ($10,000×100/90.4 = $11,602). These amounts were rounded to the nearest thousand.

Descriptive analyses were conducted to outline historical trends in self-reported dental insurance coverage, cost-barriers to dental care and out-of-pocket expenditures. These results were stratified by several socio-demographic factors (age, sex, education, employment characteristics, etc.). Comparisons were made between trends for middle-income earners to other income levels (i.e. lowest, lower-middle, higher-middle, and the highest income levels) and to national averages.

SPSS 18.0 was used for the analysis of data. Sample weights as calculated by Statistics Canada were applied to all data, where applicable. Data were displayed graphically for the years where information was available nationally. Additionally, indexes of change were calculated using the following formula: the value of each of the years in a time period/beginning year's value x 100. According to the Organization for Economic Co-operation and Development (OECD), an index of change can help to highlight change in a series from one period to another [19].

## Results

### Self-reported dental insurance coverage

**Figure 1:**
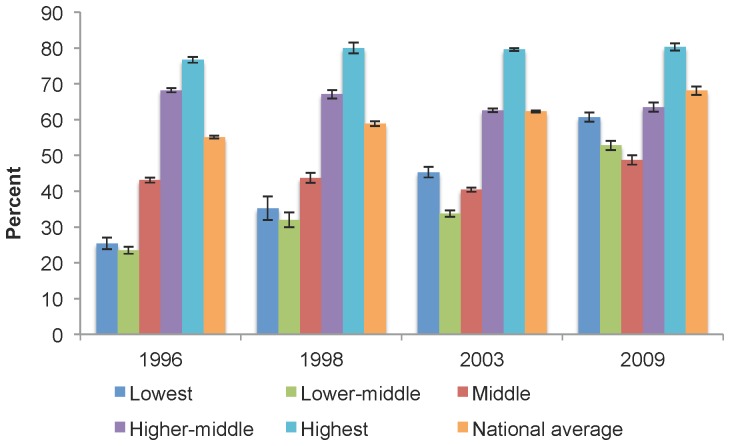
Dental insurance coverage in Canada, by income adequacy and the national average, select years. The proportion of middle-income Canadians with dental insurance increased from 43.1% in 1996 to 48.7% by 2009. By 2009, middle-income earners had the lowest levels of dental insurance coverage when compared to all other income groups. While the national average rose steadily between 1996 and 2009, from 55.1% to 68.1%, the proportion of insured middle-income earners declined from 43.1% in 1996 to 40.4% by 2003, then increased to 48.7% by 2009.

Figure 1 shows self-reported dental insurance coverage in Canada by income adequacy for select years. As can be seen, the proportion of middle-income Canadians with dental insurance increased from 43.1% (95% CI: 42.4, 43.9) in 1996 to 48.7% (95% CI: 47.4, 50.0) by 2009. Importantly though, by 2009, middle-income earners had the lowest levels of dental insurance coverage when compared to all other income groups. Figure 1 also compares these results to national trends in dental insurance. While the national average rose steadily between 1996 and 2009, from 55.1% (95% CI: 54.7, 55.5) to 68.1% (95% CI: 66.9, 69.3), the proportion of insured middle-income earners declined from 43.1% (95% CI: 42.4, 43.9) in 1996 to 40.4% (95% CI: 39.8, 41.0) by 2003, then increased to 48.7% (95% CI: 47.4, 50.0) by 2009.

**Figure 2:**
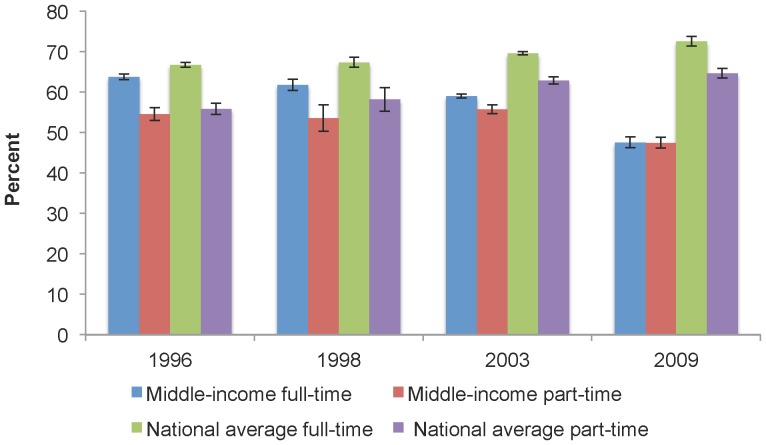
Dental insurance coverage, middle-income and the national average, by work status, select years. When compared to national averages, middle-income earners had lower levels of dental insurance coverage for both full- and part-time workers. Middle-income full- and part-time workers also had the same level of dental insurance in 2009 (47.6% vs. 47.5%), yet nationally, for all incomes, a difference was seen between the two, with a greater percentage of full-time workers having dental insurance compared to their part-time counterparts (72.6% vs. 64.7%).

Figure 2 shows dental insurance coverage levels among middle-income full-time and part-time workers compared to the national averages for these groups. When compared to national averages, middle-income earners had lower levels of dental insurance coverage for both full- and part-time workers. Middle-income full- and part-time workers also had the same level of dental insurance in 2009 (47.6% (95% CI: 46.3, 48.9) vs. 47.5% (95% CI: 46.2, 48.8)), yet nationally, for all incomes, a difference was seen between the two, with a greater percentage of full-time workers having dental insurance compared to their part-time counterparts (72.6% (95% CI: 71.4, 73.8) vs. 64.7% (95% CI: 63.5, 66.0)).

### Self-reported cost-barriers to dental care

**Figure 3:**
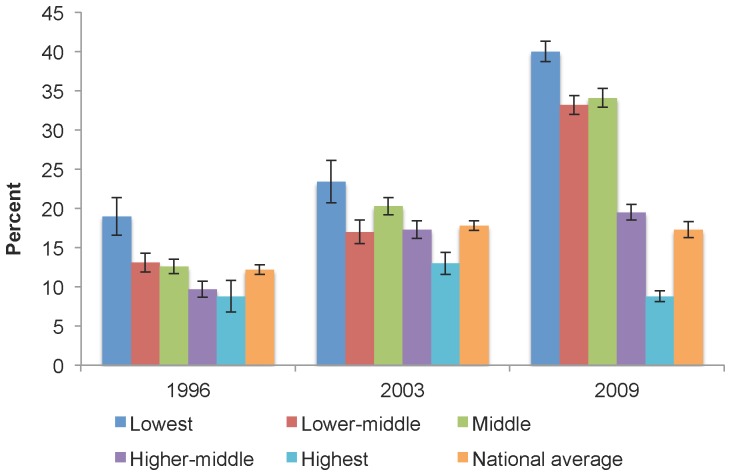
Self-reported cost-barriers to dental care, by income adequacy the national average, select years. The percentage of middle-income Canadians who reported cost-barriers increased from 12.6% in 1996 to 34.1% by 2009. When comparing the percentage of middle-income Canadians reporting cost-barriers to the national average, a general increase can be seen in both groups from 1996 to 2009. However, the level of growth is much higher among the middle-income group.

National data on perceived cost-barriers to dental care were only available for the years 1996, 2003 and 2009. Figure 3 illustrates the percentage of Canadians who reported financial barriers to dental care in select years as a national average and stratified by income adequacy. The percentage of middle-income Canadians who reported cost-barriers increased from 12.6% (95% CI: 11.7, 13.7) in 1996 to 34.1% (95% CI: 32.9, 35.3) by 2009. When compared to all other income groups, middle-income Canadians had the largest rise in the levels of such reports with an index of change of 270.6 by 2009 (data not shown). When comparing the percentage of middle-income Canadians reporting cost-barriers to the national average, a general increase can be seen in both groups from 1996 to 2009. However, the level of growth is much higher among the middle-income group. While in 1996 the level reporting cost-barriers among middle-income Canadians was only slightly different from national levels (12.6% (95% CI: 11.7, 13.7) vs. 12.2% (95% CI: 11.6, 12.8)), by 2009 middle-income Canadians made such reports about twice more than the national average (34.1% (95% CI: 32.9, 35.3) vs. 17.3% (95% CI: 16.3, 18.3)). Importantly, between 2003 and 2009 when reports made by middle-income Canadians increased from 20.3% (95% CI: 19.2, 21.5) to 34.1% (95% CI: 32.9, 35.3), the national average remained about the same (17.8% (95% CI: 17.2, 18.4) to 17.3% (95% CI: 16.3, 18.3)).

**Figure 4:**
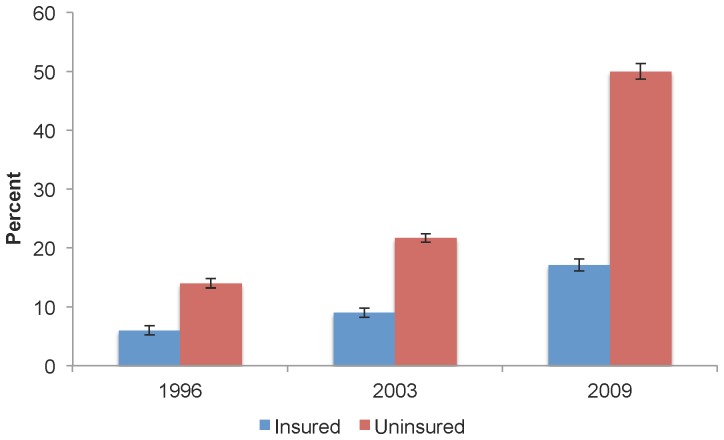
Self-reported cost-barriers to dental care among middle-income Canadians, by insurance status, select years. While the proportion of insured persons reporting cost-barriers to dental care grew from 6.0% in 1996 to 17.1% by 2009, the proportion of uninsured persons who made such reports rose from 14.0% to 50.0%.

Major differences were also observed in self-reported cost-barriers amongst insured and uninsured middle-income Canadians (Figure 4). While the proportion of insured persons reporting such barriers grew from 6.0% (95% CI: 5.2, 6.9) in 1996 to 17.1% (95% CI: 16.1, 18.1) by 2009, the proportion of uninsured persons who made such reports rose from 14.0%, (95% CI: 13.2, 14.9) to 50.0% (95% CI: 48.7, 51.3). As represented by the indexes of change (data not shown), the percentage of cost-barrier reports grew the fastest among older adults (index of change = 734.4), middle-aged adults (index of change = 366.1), and the uninsured (index of change = 362.3), whereas the respective rise among young adults (index of change = 140.5) and those with higher educational attainment was the lowest (index of change = 172.8).

### Expenditures for dental care

**Figure 5:**
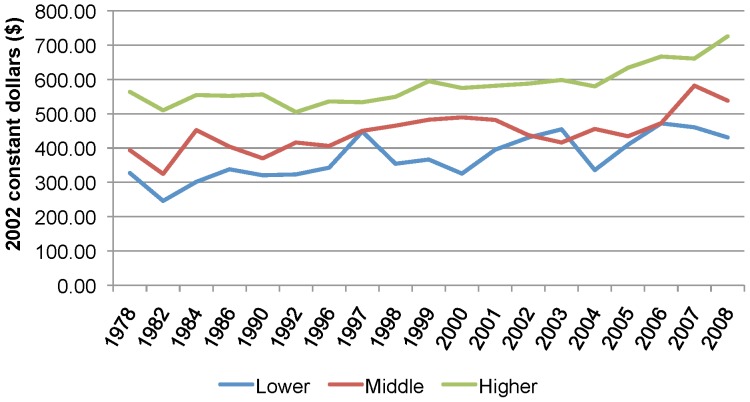
Average household expenditures for dental care in Canada, by income adequacy, 1978–2008. The average household expenditures for dental care among middle-income households grew from approximately $394 in 1978 to $538 by 2008 (2002 constant dollars). From the early 1990s to the early 2000s, dental expenditures per middle-income household began to rise faster relative to other income levels. By 2003, lower-income households reported more expenditures per household compared to middle-income households. Over the same period, higher-income household expenditures remained relatively stable. By 2008, middle-income households had the largest rise in out-of-pocket expenditures since 1978.

Figure 5 shows that the average household expenditures for dental care among middle-income households grew from approximately $394 in 1978 to $538 by 2008 (2002 constant dollars). From the early 1990s to the early 2000s, dental expenditures per middle-income household began to rise faster relative to other income levels with the index of change increasing from 94.0 in 1990 to 124.3 by 2000 (data not shown). By 2003, lower-income households reported more expenditures per household compared to middle-income households. Over the same period, higher-income household expenditures remained relatively stable. By 2008, middle-income households had the largest rise in out-of-pocket expenditures since 1978 (index of change = 136.7, data not shown).

## Discussion

This study is the first to explore the issue of access to dental care among middle-income Canadians. When compared to all other income levels and to the national average, this study found that, by 2009, middle-income Canadians had the lowest levels of dental insurance coverage and the greatest increase in perceived cost-barriers to dental care. By 2008, middle-income Canadians also had the greatest rise in out-of-pocket dental expenditures since 1978. As proxies of access, this study suggests that access to dental care for this segment of the Canadian population may have become more difficult over the years of observation.

### Trends in dental insurance coverage

Dental insurance coverage for those in the lowest and lower-middle income levels consistently rose from 1996 to 2009, to the point where they surpassed those of middle-income Canadians. This is likely related to the fact that since 2004, five provincial governments have introduced targeted investments in publicly financed dental care, which has resulted in more dental coverage for eligible low-income Canadians. Again, this has occurred in a context where more than half of middle-income Canadians remain uninsured, with no observed improvements in dental insurance coverage since 1996. Alongside increases to the costs and demand for dental care, and the lack of growth in Canadian incomes, it is no surprise then that middle-income Canadians report increasing difficulties in accessing dental care. In fact, recent national estimates derived from the CHMS demonstrated that middle-income Canadians are over one-and-a-half times more likely to have an unmet dental treatment need than those of the highest incomes [20], and when compared to both high- and low-income families, had the highest mean caries severity scores among their children [15].

Similar to the findings of Quiñonez and Grootendorst (2011), this study suggests that changes in the labour market have challenged Canada's middle-income sector more than the rest [1]. For example, while at the national level the proportion of insured full- and part-time workers has grown, among middle-income workers, whether full- or part-time, levels of insurance have stagnated. Also, historical literature has consistently demonstrated that a greater proportion of full-time workers nationally have dental coverage when compared to part-time workers, yet for middle-income Canadians, no difference was found between these categories. This suggests that middle-income full-time workers have experienced the most drastic changes to their workplace arrangements, which, as discussed in the introduction, stems from decreases to both the quality and availability of employment-based dental insurance. These findings can be relevant to other countries as well, particularly the United States, considering that access to dental care is very similar in both countries given the significant similarities in dental care systems, both of which rely heavily on the presence of employment-based dental insurance. Again, due to economic changes, the labour market in the United States has also shifted towards more non-standard jobs, which has resulted in the decline of employment-based insurance for full-time workers from 77% in 1984 to 57% by 1995 [21]. Reports of unmet needs for dental care among middle-income Americans have also increased from 3.9% in 1999 to 5.4% by 2002 [14].

### Trends in cost-barriers to dental care

Similar to recent work by Thompson (2012), this study found that those from the lower and lower-middle income groups reported the greatest cost-barriers to dental care [22]. However, from 1996 to 2009, the proportion of middle-income Canadians who reported such barriers surpassed that of the lower-middle income group, increasing by approximately three times, resulting in the highest increase among all income levels. This again suggests that changes to dental care plans seen within this time period have had an impact. This is further supported by Thompson (2012), who noted that, while middle-income earners were generally able to access dental professionals, they reported the greatest levels of declining recommended dental treatment due to cost [22].

This study also found that by 2009, three times as many uninsured middle-income Canadians reported cost-barriers to dental care compared to their insured counterparts. This again confirms the fundamental role of dental insurance in accessing dental care in terms of how it mitigates the potential barrier of upfront costs [2], [7], [22]. Thompson (2012) found that after controlling for other factors, including income, uninsured Canadians were almost six times more likely to report cost-barriers to dental care when compared to their insured counterparts [22].

Importantly, it appears that middle-income seniors also experienced the greatest rise in cost-barriers to dental care. This is not surprising considering that older adults are retaining higher numbers of teeth, thereby potentially increasing their dental needs at a time when they may also be experiencing the diminished income and loss of dental insurance coverage associated with retirement [23]. To be sure, the CHMS found the highest levels of dental disease and the highest rate of no insurance among adults 60–79 years of age [15]. It has also been found that middle-income seniors are the most affected in retirement by the loss of their employment-based dental insurance, as unlike upper-income seniors, they lack sufficient disposable income to afford the cost of dental care [23].

### Trends in expenditures for dental care

In the absence of adequate dental coverage, individuals are required to spend more money out-of-pocket on dental care (if they ultimately decide to seek care) [22], [24]. In this way, out-of-pocket expenditures on dental care can act as a reasonable proxy of access, especially in the context of an insurance rich market such as Canada's, meaning the more an individual has to spend out-of-pocket, the more difficult it may actually be to obtain care [1], [25]. This study found a general increase in average out-of-pocket expenditures on dental care from 1978 to 2008 among all income levels. As Quiñonez and Grootendorst (2011) have suggested, this is arguably a result of a decline in the robustness of current dental plans, overall increases in dental prices well beyond inflation, and the shift toward demanding more expensive services [1]. Again, the most drastic increases in out-of-pocket expenditures for dental care were found among middle-income Canadians. This study also showed that the average expenditures for middle- and low-income households were relatively volatile, while the respective changes for high-income households were far steadier.

### Limitations

This study relied heavily on the availability of consistent data. Although most of the outcomes and socio-demographic variables used were collected reliably over the years as well as between surveys, there were some instances of inconsistency. For example, the years 2005 and 2007 of the CCHS did not provide income adequacy data and the categorical income variable available did not allow for recoding into the desirable format. Dental insurance data was also only collected for some provinces. Therefore, to be more generalizable, these years were excluded. Also, since the current study identified trends among the target population, the results do not provide information on cause-and-effect relationships or statistically significant differences. Finally, although this study focused on affordability as proxy of access, it is acknowledged that the concept of access encompasses many other factors, including geographic and cultural considerations.

### Policy implications

It can be argued that middle-income Canadians are now facing affordability issues in accessing care comparable to those historically reported by only the lowest income earners. This is of concern, especially since middle-income Canadians have never been on the policy agenda for public assistance to this point. That said, from a public health perspective, some clarity is needed on exactly what is cost-prohibitive, orthodontic and cosmetic dental care, or basic fillings and extractions. Each has its own implications.

It is also clear that changes in dental care financing are important for the ability of households to access dental care. Hence, as a policy instrument, removing the price barrier to care is likely of most importance [1], [26]. As suggested by Thompson (2012), the eligibility criteria for public dental insurance coverage could be altered to include segments of the middle-income population, and/or policies could be made to increase non-wage offers from employers, or to promote the enrolment of workers in employment-based dental insurance plans overall [22]. These policies may, for example, include increasing tax benefits for employers, or mandating the presence of health care benefits in all employment-employee contracts [22]. Whichever way, getting more middle-income earners insured will help to alleviate their cost-barriers to dental care through a reduction in costs at point of purchase.

## Conclusions

This study suggests that affordability issues in accessing dental care are no longer just a problem for the lowest income groups in Canada, but now involve middle-income earners as a consequence of their lack of, or decreased access to, comprehensive dental insurance.
